# Primary peritoneal carcinoma with late-phase pulmonary metastases: a case report

**DOI:** 10.1186/s40792-019-0752-0

**Published:** 2019-12-10

**Authors:** Naoki Ozeki, Shuhei Hakiri, Hisashi Tateyama, Kohei Yokoi, Toyofumi Chen-Yoshikawa

**Affiliations:** 1 Department of Thoracic Surgery, Nagoya University Graduate School of Medicine, 65 Tsurumai-cho, Showa-ku, Nagoya, 466-8550 Japan; 2 Department of Pathology, Kasugai Municipal Hospital, 1-1-1, Takaki-cho, Kasugai-shi, 486-0000 Japan

**Keywords:** Peritoneal carcinoma, Lung, Metastasis, Fallopian tube carcinoma

## Abstract

**Background:**

Primary peritoneal carcinoma (PPC) is a very rare and aggressive type of malignancy with a poor prognosis.

**Case presentation:**

A 66-year-old woman was referred to our hospital with two pulmonary nodules that developed after PPC resection and postoperative adjuvant chemotherapy administered 5 years earlier. Computed tomography revealed a 1.3-cm-sized nodule in the left lung with a small airspace in the posterior basal segment and a 0.9-cm-sized solid nodule in the apico-posterior segment that grew slightly within a 2-month period. 18F-Fluorodeoxyglucose-positron emission tomography of these lesions revealed respective maximum standardized uptake values of 7.11 and 2.46. Her serum cancer antigen-125 level remained within the normal range, despite elevation before the first surgery. The posterior basal segment and superior division were subjected to anatomical segmentectomy. An intraoperative frozen section examination could not distinguish metastatic PPC from primary lung cancer. Immunopathologically, the two nodules were identified as metastatic PPC.

**Conclusions:**

Our findings suggest that PPC patients may develop late-phase thoracic recurrence that is difficult to diagnose clinically after initial treatment in a potentially resectable setting.

## Introduction

Primary peritoneal carcinoma (PPC) is a rare type of malignancy that is clinically and histopathologically indistinguishable from ovarian serous or primary fallopian tube carcinoma [1, 2]. Currently, these high-grade serous carcinomas may have similar clinical presentations and courses. However, PPC patients generally present with a more advanced disease stage and have poorer prognosis [1–3]. All of them may arise from serous tubal intraepithelial carcinomas (STICs) at the distal fimbriated end of the fallopian tube [1, 2]. PPC incidence is very low; therefore, only a few thoracic metastasis cases have been reported. Here, we report a rare case of pulmonary metastasis from PPC that arose 5 years after the initial surgery in a potentially resectable setting.

## Case report

**Fig. 1:**
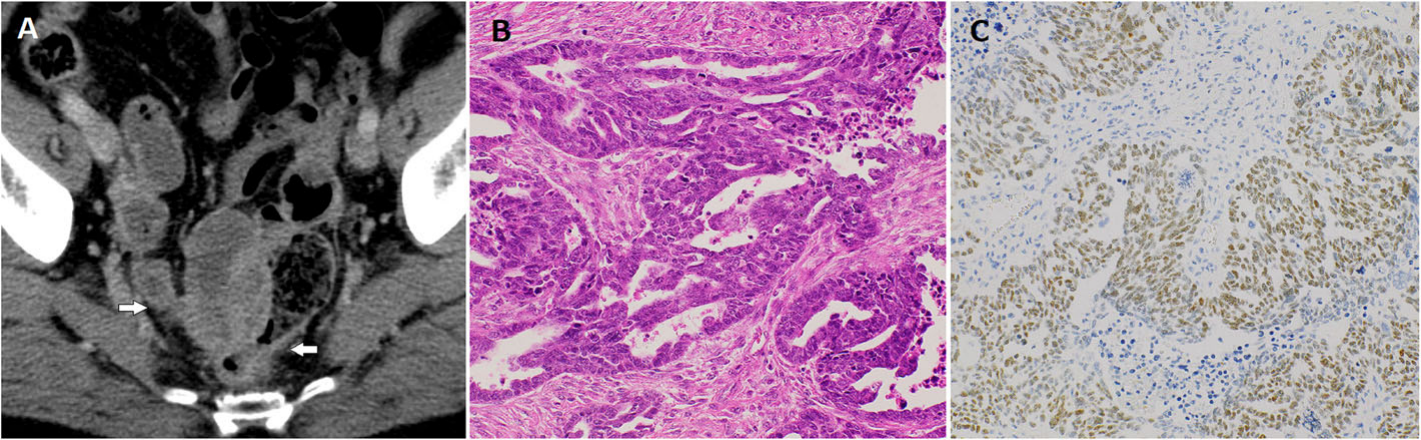
Contrast-enhanced computed tomography showing a pelvic peritoneal mass with irregular enhancement in the neighboring ileum and rectum. Ovaries and regional lymph nodes were not involved (**a**). Histological examination revealed irregular clusters of malignant cells with slit-like glandular spaces (magnification × 200) (**b**). Immunohistochemically, the tumor was positive for Wilms tumor-1 (× 200) (**c**)

A 66-year-old woman was referred to our hospital with two pulmonary nodules that developed after PPC resection and subsequent adjuvant chemotherapy 5 years earlier. She underwent ileocecal resection and low anterior resection of the involved rectum, which yielded a 6.0-cm-sized PPC mass (Fig. 1a). The mass was initially considered mesenteric, and the lack of ovarian or uterine abnormalities or involvement on preoperative radiological examinations and intraoperative findings led to a strong suspicion of a gastrointestinal stromal tumor of the ileum. The distal end of the right fallopian tube was resected because of tumor involvement. The ovaries and uterus were considered normal and thus were not resected. Histopathologically, the tumor mass comprised irregular clusters of malignant cells with cellular papillae or slit-like glandular spaces (Fig. 1b), with or without psammoma bodies. The tumor cells contained atypical large nuclei, prominent nucleoli, and frequent mitoses. Immunohistochemically, these cells were positive for Wilms tumor (WT)-1 (Fig. 1c), cytokeratin (CK) 7, paired box gene 8, and estrogen receptor and negative for CK20, caudal-type homeobox 2, and villin. The mass was diagnosed as PPC based on the diagnostic criteria of the Gynecologic Oncology Group [1, 2]. Ascites and lymph node metastasis were not observed. Thus, tumor resection was considered complete (R0). She underwent six adjuvant chemotherapy cycles with paclitaxel (175 mg/m^2^) and carboplatin [area under the curve (AUC): 5].

**Fig. 2:**
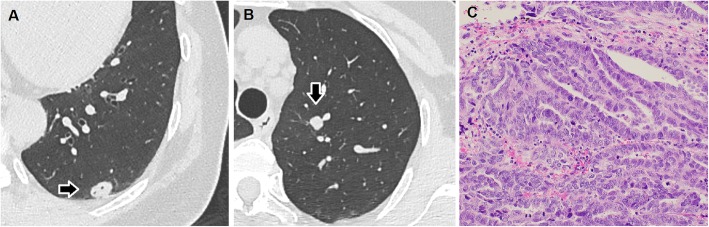
Chest computed tomography showing a 1.3-cm-sized nodule with small airspace in the left posterior basal segment (**a**) and a 0.9-cm-sized solid nodule in the left apico-posterior segment (**b**). A histologic examination of the resected lung specimen resembling the previous primary peritoneal carcinoma. Irregular clusters of malignant cells with slit-like glandular spaces were observed. The tumor cells contained atypical large nuclei, prominent nucleoli, and frequent mitoses (× 200) (**c**)

Follow-up computed tomography showed a 1.3-cm-sized nodule in the left lung with a small airspace in the posterior basal segment (Fig. 2a) and a 0.9-cm-sized solid nodule in the apico-posterior segment that grew slightly during a 2-month period. No pleural effusion was detected. 18F-Fluorodeoxyglucose (FDG)-positron emission tomography (PET) revealed maximum standardized uptake values of 7.11 and 2.46 for these nodules. Both nodules were suspected to be pulmonary metastases of PPC. However, the serum cancer antigen (CA)-125 level was within normal range (11.7 U/ml). Because the serum CA-125 level was elevated (529.1 U/ml) before the first surgery, primary lung cancer or non-malignancy was also suspected. No evidence of tumor in the primary site was observed on FDG-PET or abdominal computed tomography.

Pulmonary wedge resection is now considered a standard surgical procedure for the treatment of metastasis from cancers in other organs. Therefore, if an intraoperative frozen examination could diagnose the nodules as metastatic PPCs, we would select wedge resection. As a result, the intraoperative frozen section examination of the resected lung specimen could not distinguish metastatic PPC from primary lung adenocarcinoma. Regarding surgical treatment of small-sized primary lung cancer, anatomical segmentectomy is gaining popularity as a curative and less-invasive procedure [4]. Accordingly, even if the two pulmonary nodules were primary lung cancers, not pneumonectomy (double lobectomies), but the respective segmentectomies would be suitable owing to the invasiveness.

From all the above, regardless of the definitive diagnosis (metastatic PPC or primary lung cancer), anatomical segmentectomies of the two nodules were deemed to be suitable, considering curativity and invasivity. Anatomical segmentectomies of the posterior basal segment and superior division with systematic lymph node dissection were performed. Histological examination of the resected specimen (Fig. 2b) revealed neoplastic cells in the lung tumor. These cells contained enlarged nuclei, irregular slit-like spaces, and a papillary pattern, similar to previous PPC (Fig. 2c). Immunohistochemically, the specimen was positive for WT-1 and negative for thyroid transcription factor-1. These findings led to a diagnosis of a pulmonary recurrence of the previously resected PPC. No mediastinal or hilar lymph node metastasis was observed. The postoperative course was uneventful, and second-line chemotherapy with paclitaxel (175 mg/m^2^) and carboplatin (AUC 5) for six cycles was initiated.

## Discussion

The Gynecologic Oncology Group has established the following specific criteria for the diagnosis of PPC: (a) normal ovaries, (b) bulk of the tumor in the peritoneum, (c) greater extraovarian involvement than ovarian surface involvement, (d) ovarian involvement limited to the surface epithelium or cortical stroma with a tumor size < 5 mm, and (e) pathological characteristics similar to those of ovarian serous cancers [1, 2]. PPCs are histologically and clinically identical to ovarian serous carcinomas and primary fallopian tube carcinomas but are generally detected at a more advanced stage and are associated with a worse poorer prognosis. Eltabbakh et al. reported that the median overall survival of PPC patients after the first surgery was 23.5 months, with a 5-year survival rate of 26.5% [3]. Moreover, a multivariate analysis identified performance status and degree of primary debulking surgery as prognostic factors [3].

Both PPC and ovarian serous carcinoma may derive from occult STICs in the fimbria of the fallopian tube [1, 2]. The risk of serous PPC increases in patients with hereditary breast and ovarian cancer syndrome or BRCA1 or BRCA2 mutations [1]. In the latter patients, most high-grade serous carcinomas may develop from STICs that implant on the peritoneal or ovarian surface [1, 2]. Although the spread of STICs may lead to PPCs and ovarian serous carcinomas, primary fallopian tube carcinomas may arise from STICs retained within the fallopian tube. Besides BRCA 1/2 mutations, these high-grade serous carcinomas arise from STICs with subsequent inactivating mutations in TP53 [1, 2].

The typical pattern of pulmonary metastasis of ovarian serous carcinoma includes pleural effusions or small nodules that exhibit FDG uptake on PET (when larger than 1 cm) and may be similar to intrapulmonary lymphoid tissue [5]. Identical pulmonary metastasis patterns may be observed from PPC and primary fallopian tube carcinoma [5]. PPC metastases most frequently manifest as malignant abdominal effusion or metastatic omental involvement. Conversely, extra-abdominal metastasis, thoracic effusion, and involvement of the lung, main bronchus, and brain parenchyma rarely occur, with few cases being reported [2, 6]. To our knowledge, only one report described a resected pulmonary metastasis of PPC that developed 5 months after initial treatment and also involved malignant thoracic effusion [6]. In our case, only pulmonary nodules were observed at recurrence, with no other residual thoracic/abdominal metastasis. Compared with previously reported recurrent PPC cases, ours was distinct because of the potentially resectable setting. However, this characteristic complicated clinical diagnosis because the lesions were difficult to differentiate from primary lung cancer.

## Conclusions

We demonstrated that a late-phase pulmonary recurrence of PPC after initial treatment can occur in a potentially resectable setting.

## Data Availability

Not applicable

## Abbreviations

PPC: Primary peritoneal carcinoma
STIC: Serous tubal intraepithelial carcinomas
WT: Wilms tumor
AUC: Area under the curve
FDG: Fluorodeoxyglucose
PET: Positron emission tomography
CA: Cancer antigen
